# The Effect of Disclosure of PrEP Use on Adherence Among African Young Women in an Open-Label PrEP Study: Findings from HPTN 082

**DOI:** 10.1007/s10461-023-04175-0

**Published:** 2023-09-28

**Authors:** Geetha Beauchamp, Sybil Hosek, Deborah Donnell, Kwun C. G. Chan, Peter L. Anderson, Bonnie J. Dye, Nyaradzo Mgodi, Linda-Gail Bekker, Sinead Delany-Moretlwe, Connie Celum, for the HPTN 082 study team

**Affiliations:** 1 Department of Health Systems and Population Health, University of Washington, Seattle, WA USA; 2 Department of Psychiatry, Stroger Hospital of Cook County, Chicago, IL USA; 3 Vaccine and Infectious Disease Division, Fred Hutchinson Cancer Research Center, 1100 Fairview Ave. N., Mail Stop M2-C200, Seattle, WA 98109 USA; 4 Department of Biostatistics, University of Washington, Seattle, WA USA; 5 Department of Pharmaceutical Sciences, University of Colorado-Anschutz Medical Campus, Aurora, CO USA; 6 FHI 360, Durham, NC USA; 7 University of Zimbabwe Clinical Trials Research Centre, Harare, Zimbabwe; 8 The Desmond Tutu HIV Centre, University of Cape Town, Cape Town, South Africa; 9 Wits RHI, Faculty of Health Sciences, University of the Witwatersrand, Johannesburg, South Africa; 10 Departments of Global Health, Medicine, and Epidemiology, University of Washington, Seattle, WA USA

**Keywords:** Adherence, HIV prevention, Adolescent girls and young women, Pre-exposure prophylaxis (PrEP), Africa, Disclosure and social support

## Abstract

To develop effective PrEP adherence interventions, it is important to understand the interplay between disclosure of pre-exposure prophalxis (PrEP) use, social support, and PrEP adherence. We leveraged the HPTN 082 study conducted among 451 adolescent girls and young women (AGYW) (ages 16 to 25 years, 2016 to 2019) in South Africa and Zimbabwe. Among the 349 who had month three disclosure and PrEP adherence data, 60% (n = 206) felt supported by adults, and 89% (n = 309) disclosed PrEP use to at least one person. PrEP disclosure was not associated with increased adherence, measured by intracellular tenofovir-diphosphate concentrations in dried blood spots. Women who reported having supportive adults, and disclosed to their parents, had higher adherence at 6 months with an increase of 177 fmol/punch (95% CI 12 to 343, t = 2.11, p = 0.04). PrEP interventions that help AGYW identify supportive relationships and effectively communicate the benefits of PrEP may improve PrEP adherence.

Clinicaltrials.gov ID number: NCT02732730.

## Introduction

African adolescent girls and young women (AGYW) experience a disproportionate rate of new HIV diagnoses, which account for 25% of all new transmissions globally [1]. Adherence to highly effective oral pre-exposure prophylaxis (PrEP) to prevent HIV acquisition is challenging for many AGYW [2–6]. Factors that negatively impact PrEP adherence for young women comprise low perceived risk of HIV acquisition, intimate partner violence, side effects, depression, stigma related to disclosure of PrEP use, and the burden of taking a daily oral pill [7–14]. AGYW are in critical need of effective intervention programs to mitigate barriers to PrEP adherence. Open-label PrEP demonstration studies aimed at increasing compliance through providing PrEP drug-level feedback, short message service (SMS) reminders, peer-adherence clubs, or conditional cash incentives to motivate AGYW to take PrEP pills persistently did not demonstrate a significant improvement in PrEP adherence compared to the standard of care support [4, 5, 15, 16]. 

Young women are at least twice as likely to acquire HIV as age-matched African men [17]. Support from social networks (e.g., parents, partners, and friends) has a crucial role in adolescents’ health-promoting behaviours, including adherence to treatment and medication as prescribed [3, 18–20]. For adolescents, their most influential relationships are parental figures, peers, and intimate partners. On the other hand, they have an increased desire for independence from parents during emerging adulthood and are prone to peer pressure and behaviorally vulnerable to HIV (e.g., sexual debut at a younger age, unprotected sex, older sexual partners, concurrent sexual partners, and alcohol abuse) [4, 13, 17–20]. Youth also lack financial autonomy and problem-solving skills, putting them at greater risk of PrEP nonadherence [21]. In addition to social support for health-promoting behaviours, young women may benefit from PrEP adherence support to prevent HIV acquisition. However, to receive support for PrEP adherence, AGYW need to weigh the benefits and risks of disclosure of PrEP use [22, 23]. In the open-label HIV Prevention Trials Network (HPTN) 067/ADAPT study in Cape Town, South Africa, and the multi-site Microbicide Trials Network (MTN) 003/VOICE trial in South Africa, Uganda and Zimbabwe, women who participated in semi-structured interviews highlighted that societal stigma made it difficult to rely on their social network for PrEP adherence support [9, 24]. Qualitative studies indicated that disclosure of PrEP use to their sexual partner can affect PrEP adherence positively (e.g., medication reminders, a place to store pills, and moral support) or negatively (e.g., mistrust, disapproval, and conflicts) due to stigma [7, 23, 25].

Young women have also reported that they consulted older female figures about PrEP because they did not trust the information from their peers and feared gossip among peers and being judged immoral [26, 27]. This suggests that the quality of the social relationship is likely to moderate the effect of disclosure of PrEP use, as is observed with antiretroviral treatment (ART) adherence [28]. Prior research indicates that supportive adult relationships, PrEP disclosure, and PrEP support from trusted members of social groups are essential components for successful PrEP adherence [7, 29, 30]. Given the stigma and gender disparities, a greater understanding of the pathways between disclosure of PrEP use, supportive adult relationships, and PrEP support received from interpersonal relationships is needed to design impactful intervention programs to assist AGYW with improving PrEP adherence.

Based on the Information, Motivation, and Behavioral model [31, 32] situated in the socio-ecological model framework [33, 34], we conceptualized that disclosure to close relationships in their social network and receiving adherence support, such as reminders to take the medication as prescribed, can influence AGYW's ability to take PrEP pills consistently [25, 26]. There is some qualitative evidence on the interplay between disclosure of PrEP use, social support, PrEP support from interpersonal relationships, and PrEP adherence. While qualitative studies provided potential hypotheses for further investigation, it not easy to determine the sequence of effect from disclosure to PrEP support to adherence, and qualitative data do not provide information on the magnitude of the impact at months three and six. We hypothesized that AGYW who disclosed PrEP use would likely have a higher PrEP adherence at months three and six and explored the potential moderating effect of supportive adult relationships. We also hypothesized that the effect of PrEP disclosure on adherence was partially mediated through reminders from those to whom they disclosed PrEP use. Therefore, our study quantitatively investigated: (1) the impact of disclosure of PrEP use on PrEP adherence by relationship type (parents, partners, and friends) at months three and six, (2) whether having supportive adults in their life moderates the magnitude of the effect on AGYW’s PrEP adherence, and (3) if PrEP support in the form of reminders to take PrEP pills mediates the effect of disclosure on adherence.

## Methods

### Study Design and Population

The HPTN 082 study, an open-label oral PrEP demonstration trial, was conducted from October 2016 to October 2019 among 451 AGYW aged 16 to 25 who are living without HIV. The study participants were recruited from youth-friendly clinics in Harare, Zimbabwe, and Cape Town and Johannesburg, South Africa. The study design, study procedures, and primary findings were published elsewhere [4]. Briefly, all study participants were offered oral PrEP at enrollment and counselled to take it daily. Those who did not initiate PrEP could start PrEP at any time up to 48 weeks during the 52 week follow-up period. The AGYW who accepted PrEP were randomized 1:1 to standard of care or enhanced adherence support. Standard of care adherence support included weekly two-way text messages during the first three months, brief counselling (months one, two, and then quarterly), and offer of participation in peer-led PrEP adherence clubs. The enhanced adherence support included PrEP adherence drug-level feedback at months two and three follow-up visits based on intracellular tenofovir-diphosphate (TFV-DP) concentration in dried blood spots (DBS) for the previous month, In addition to standard of care adherence support. The follow-up visits were at 4, 8, and 12 weeks, and then quarterly. The participants were given 30-day supplies of PrEP pill through the first 12 weeks and then increased to a 3-month supply during the follow-up visits from months three through 12.

### Data Collection

The study staff collected demographic data, including age, education, and housing status, on the case report form at enrollment. A self-administered computer-assisted self-interview (CASI) questionnaire was used to obtain sensitive data, including HIV likelihood perception, sexual behavior, and depressive symptoms at enrollment and then quarterly for up to 12 months. PrEP use, disclosure of PrEP use, and PrEP reminder data were collected quarterly by CASI from participants who accepted PrEP.

#### Measures

##### PrEP Adherence

The outcome measurement was PrEP adherence at months three and six, assessed using intracellular TFV-DP concentration in DBS, which provides an objective biomarker measure of average PrEP adherence in the prior 4–6 weeks [35]. A DBS concentration of TFV-DP ≥ 700 fmol/punch is associated with taking an average of four or more doses per week, which provided 96% risk reduction among men who have sex with men (MSM), and 200 fmol/punch of TFV-DP is associated with about one dose per week among MSM [36, 37]. The limit of detection for TFV-DP concentration was 33.4 fmol/punch, and non-quantifiable values were set to half of the limit of detection (16.7 fmol/punch).

##### Disclosure of PrEP Use

The primary exposure *disclosure of PrEP use* was assessed using the question ‘Have you told *anyone* that you are taking PrEP?’ asked three months after starting PrEP. If the response was ‘yes’, then relationship-specific (parents, sex partners, and friends) questions were asked. The relationship types were not mutually exclusive. We assigned ‘no’ for the relationship-specific disclosure questions if the AGYW responded ‘no’ to *anyone*.

##### Support

To assess if supportive adult relationships modified the effect of disclosure of PrEP use on adherence, we used the question, ‘In general, how supported do you feel by the adults in your life?’ asked at enrollment. ‘In general, how supported do you feel by your close friends in your life?’ was used to evaluate proportion of AGYW who felt supported by close friends. The responses were on a 3-point Likert scale. We dichotomized as *well-supported* if the response was ‘very well supported’ and *not well-supported* if it were ‘almost never supported’ or ‘sometimes supported’.

##### PrEP Support

In this paper, we defined PrEP support as 'a reminder to take PrEP'. We used two questions that were asked three months after initiating PrEP, and they were ‘My family and friends who know I am on PrEP help me remember to take PrEP’ and ‘My household members who know I am on PrEP help me remember to take PrEP’. The responses were on a 5-point Likert scale [0 to 4] from ‘strongly disagree’, ‘disagree’, ‘neither disagree or agree, ‘agree’, to ‘strongly agree’.

##### HIV Likelihood Perception, Transactional Sex, Condom Use and Depression

We used ‘How would you describe your chances of getting HIV in the next year’ to evaluate HIV likelihood perception. It was dichotomized as *perceived HIV likelihood next year* if responses were small, moderate, great chance, and prefer not to answer. Transaction sex was defined as having sex with a man in exchange for food, clothes, cosmetics, transportation, items for children, and other items. 'Prefer not to answer' responses were categorized as condomless sex. A brief version (10-item) of the Center for Epidemiologic Studies Depression scale (CESD-10) was used to assess depressive symptoms [38, 39].

### Statistical Analysis

Baseline characteristics were summarised by PrEP use disclosure status at month three. The Kruskal–Wallis test was used to test group differences for continuous variables and the Pearson Chi-squared test for categorical variables. We used linear regression to assess the association between PrEP adherence and disclosure of PrEP use to *anyone*, and each of the social relationships: parents, sex partners, and friends, adjusted for age (adherence increases with age) [40–43] and the three enrollment sites to adjust for social and economic differences among the sites [1]. To evaluate whether the effect of disclosure of PrEP use on adherence was modified by AGYW who had versus did not have supportive adults, we repeated the analysis separately for those who were *well-supported* and *not well-supported*.

**Fig. 1:**
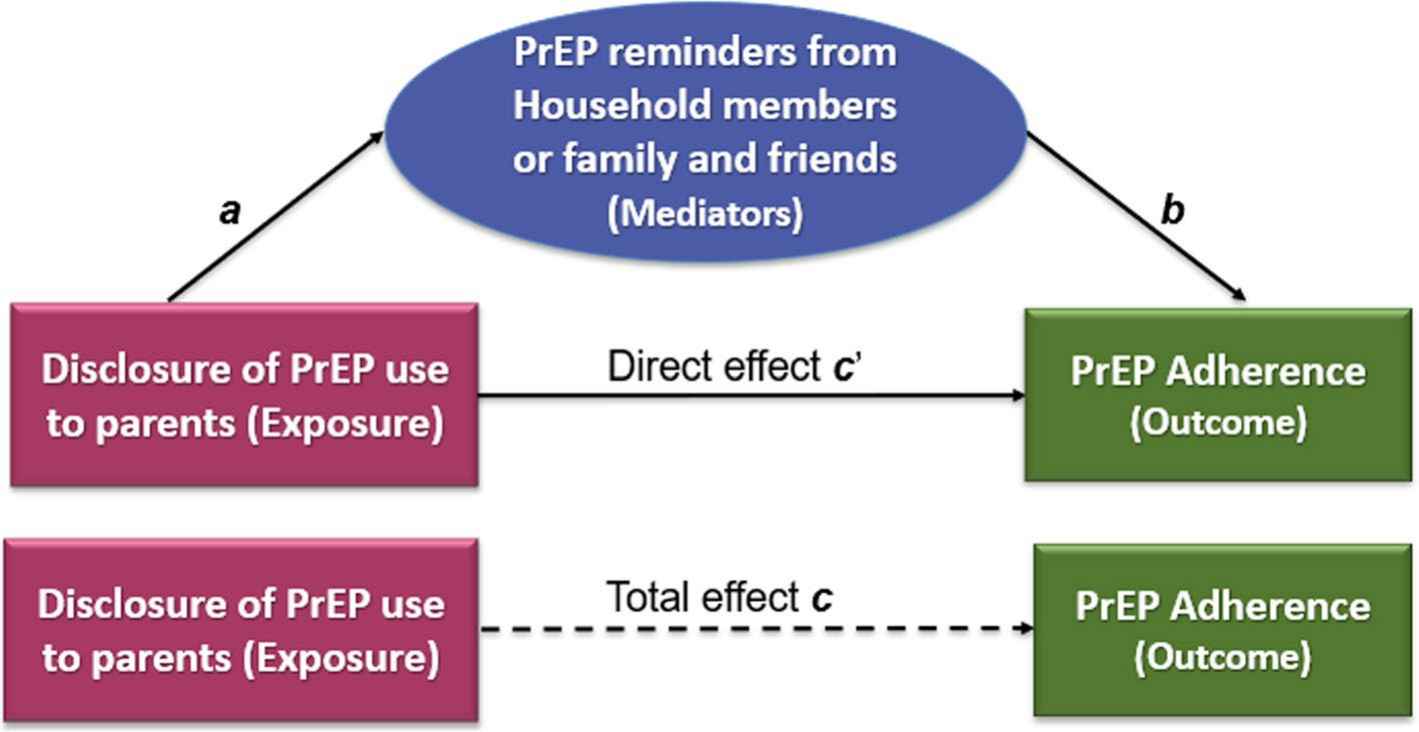
Diagram of mediation analysis showing the effect of disclosure of PrEP use on PrEP adherence mediated through PrEP reminders. The product (a*b) of the coefficients of *reminder to take PrEP* is the indirect effect, and the direct effect is the coefficient of the *disclosure of PrEP use* adjusted for the reminders to take PrEP

We conducted mediator analyses to assess if PrEP reminders were in the pathway between disclosure of PrEP use and adherence. The total effect (***c*** in Fig. 1) of disclosure of PrEP use on adherence is decomposed as direct effect (***c’***) and indirect effect (paths *a* and *b*). The total effect equals the sum of indirect and direct effects when all three regression analyses were conducted on data with complete cases for exposure, outcome, mediator, and adjusted for covariates (age and sites). First, to assess the effect of disclosure (***a***) on PrEP reminders (mediator), we regressed reminder to take PrEP on disclosure of PrEP use (exposure). Next, we regressed PrEP adherence (outcome) on disclosure of PrEP use adjusted for the reminders to take PrEP. The effect of the PrEP reminder on adherence is denoted by path ***b***. The product of the coefficients of *reminder to take PrEP* of the first and the second models (a*b) is the indirect effect, and the direct effect (***c’***) is the coefficient of the *disclosure of PrEP use* in the second model [44].

#### Post-Hoc Analysis: Living with Parents

Based on the results of the initial analysis and recent literature where adherence was significantly higher among AGYW ≤ 18 years old who disclosed PrEP use to their parents, we conducted a post-hoc analysis to investigate if there was differential PrEP support that contributed to higher adherence for AGYW who disclosed to their parents and also lived with them versus those who lived alone or with others. We used the question ‘With whom do you live?’, which had ‘mark all that apply’ response to identify AGYW who lived with their parents. The response choices were partner, parents(s), sibling(s), alone, with own children, roommate(s), and other. AGYW were considered living with their parents regardless of if they also marked other options. We created three levels of disclosure of PrEP use by living status: (1) disclosed to parents and lived with them; (2) disclosed to parents but did not live with them; and (3) did not disclose to parents. All analyses were conducted using SAS version 9.4 (SAS Institute, Cary, NC).

## Results

Of the 427 AGYW who accepted PrEP, 371 (87%) had month three PrEP adherence biomarker (DBS TFV-DP) measures and 349 (82%) had data about their disclosure of PrEP use; 343 (80%) had complete data, and were included in the analysis. Overall, the median age was 21 years [Interquartile range (IQR): 19 to 22], 20% were ≤ 18 years of age, 52% lived with their parents, and 23% lived with their partner (Table 1). Eighty-five percent had a primary sex partner, and 50% had a primary partner living with HIV or whose HIV status was unknown. Although the characteristics were similar between those who disclosed PrEP use to someone compared to those who did not, intimate partner violence was higher among AGYW who did not disclose PrEP use to anyone than among AGYW who disclosed PrEP to at least one person (63% versus 46%, χ^2^ = 3.97, p = 0.05).

**Table 1:** Baseline characteristics of the participants by disclosure of PrEP use to anyone

Baseline characteristics	Overall	Disclosed to anyone by month three		
N (%) or (median, IQR)	N = 349	Yes N = 309	No N = 40	χ^2^ (P value)
Age, median (years)	21 (19, 22)	21 (19, 22)	21 (20, 23)	1.94 (0.16)^a^
Age $\le$ 18 years	69 (20%)	63 (20%)	6 (15%)	0.65 (0.42)
Secondary school or higher	204 (58%)	181 (59%)	23 (58%)	0.02 (0.90)
Intimate partner violence, past year	166 (48%)	141 (46%)	25 (63%)	3.97 (0.05)
Perceived HIV likelihood in the next year^c^	182 (52%)	159 (52%)	24 (60%)	1.00 (0.32)
Primary sexual partner, past 3 months	296 (85%)	262 (85%)	34 (85%)	0.01 (0.94)
Primary partner living with HIV or unknown HIV status	176 (50%)	155 (50%)	21 (53%)	0.08 (0.78)
More than one partner, past year	44 (13%)	41 (13%)	3 (8%)	0.55 (0.45)^b^
Transaction sex, last 3 months^d^	85 (24%)	76 (25%)	9 (23%)	0.08 (0.65)
Always used condom with vaginal sex^e^	62 (18%)	55 (18%)	7 (18%)	0.00 (1.00)
Depressive symptom CES-D ≥ 10^f^	171 (49%)	152 (49%)	19 (48%)	0.04 (0.84)
Living with parents	180 (52%)	159 (52%)	21 (53%)	0.01 (0.92)
Living with partner	80 (23%)	71 (23%)	9 (23%)	0.00 (0.94)

^a^The median and interquartile range (IQR) are provided for continuous variables

^b^Fisher's exact two-sided p value

^c^'How would you describe your chances of getting HIV in the next year' was dichotomized as *perceived HIV likelihood next year* if responses were small, moderate, great chance and prefer not to answer

^d^Transaction sex is defined as having sex with a man in exchange for food, clothes, cosmetics, transportation, and items for children, and other items

^e'^Prefer not to answer' responses were categorized as condomless sex

^f^Center for Epidemiologic Studies Depression scale is the sum of 10 items (CESD-10), and the range is 0 to 30. CESD-10 score ≥ 10 indicates a likelihood of depressive symptoms

Three months after starting PrEP, 89% (n = 309) of AGYW disclosed PrEP use to at least one person: 72% (n = 251) reported disclosing to their parents, 76% (265) to their sex partners, and 82% (287) to their friends (Table 2). By month six, the cumulative number of AGYW who disclosed PrEP use to anyone increased by only a percent from month three. The average TFV-DP concentration among those who disclosed to anyone, parents, sex partners, or friends were comparable. The DBS TFV-DP ranged from 431 to 441 fmol/punch at month three and 363 to 381 fmol/punch at month six. Drug concentration at month three signals PrEP uptake, and month six indicates persistent adherence. When comparing the impact of disclosure to anyone versus no one on PrEP adherence, we observed a nonsignificant decrease (− 18 fmol/punch, 95% confidence interval (CI) − 153 to 118, t = − 0.28, p = 0.80) in DBS TFV-concentrations of TFV-DP at month three and an increase of 77 fmol/punch, (95% CI − 97 to 251, t = 0.87, p = 0.38) at month six. Even though the association between disclosure to parents, sex partners, or friends and PrEP adherence was nonsignificant, AGYW who disclosed PrEP use to their parents had modestly higher adherence with an increase of TFV-DP concentration of 84 fmol/punch (95% CI − 16 to 184, t = 1.64, p = 0.10) at month three, and 96 fmol/punch (95% CI − 35 to 228, t = 1.46, p = 0.15) at month six compared to those who did not disclose.

**Table 2:** Association between disclosure of PrEP use by month three and PrEP adherence at months three and six

Disclosed PrEP use by 3 months		Increase DBS TFV-DP							
		Month three adherence^a^				Month six adherence^a^			
	N = 349	Mean (95%CI)	fmol/punch (95% CI)	T test	P value	Mean (95%CI)	fmol/punch (95% CI)	T test	P value
Anyone	309 (89%)	431 (387, 474)	− 18 (− 153, 114)	− 0.28	0.78	368 (307, 429)	77 (− 97, 251)	0.87	0.38
Parents	251 (72%)	441 (401, 497)	84 (− 16, 184)	1.64	0.10	375 (310, 440)	96 (− 35, 228)	1.46	0.15
Sexual partners	265 (76%)	434 (387, 482)	8 (− 96, 113)	1.16	0.88	381 (314,448)	102 (− 34, 237)	1.46	0.15
Friends	287 (82%)	436 (391, 481)	21 (− 98, 141)	0.33	0.74	363 (300, 427)	56 (− 102, 215)	0.69	0.49

*DBS TFV-DP* tenofovir-diphosphate concentrations in dried blood spots, *CI* confidence interval

^a^Adjusted for site and age

**Fig. 2:**
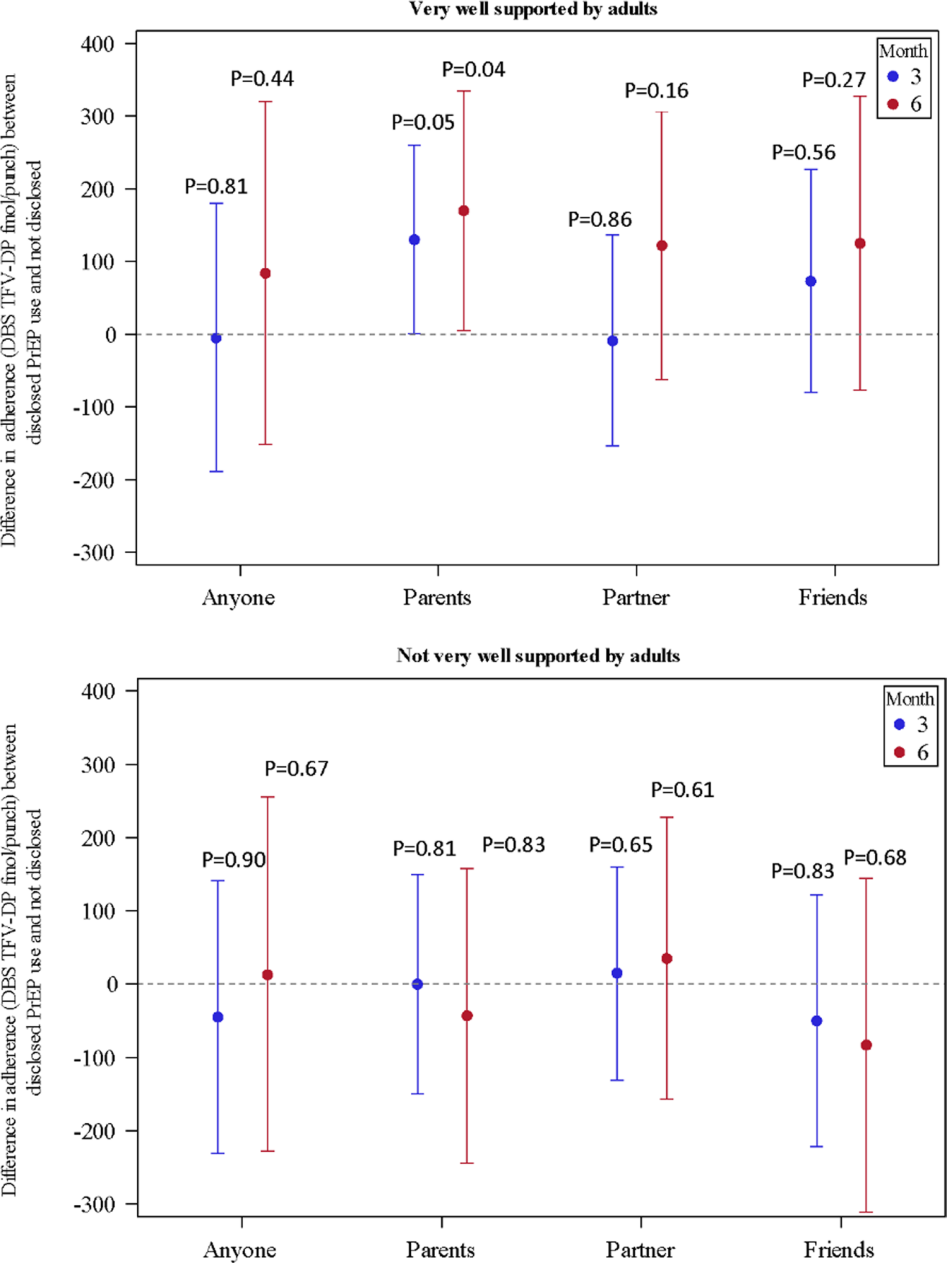
Differences in adherence between AGYW who disclosed PrEP use compared to non-disclosure and by whether AGYW reported supportive adults. Note that vertical lines represent 95% confidence levels

At study entry, 60% (n = 206) of the AGYW reported they felt well supported by the adults in their lives, while 42% (n = 145) felt well supported by their friends. Among the AGYW who had supportive adults, those who disclosed PrEP use to their parents had significantly higher PrEP adherence at month six with an increase of TFV-DP concentration of 177 fmol/punch (95% CI 12 to 343, t = 2.11, p = 0.04), compared to those who did not disclose to their parents (Fig. 2). In contrast, AGYW who did not have supportive adults had slightly lower adherence at month six with a decrease of TFV-DP concentration 22 fmol/punch (95% CI − 232, 187, t = − 0.21, p = 0.83) among those who disclosed to their parents compared to those who did not disclose PrEP use.

There was no association between disclosure of PrEP use to anyone and each of the social relationships and reminders to take PrEP by household members or family and friends, except disclosure to parents was significantly associated with an increase in the PrEP reminder score for household members (0.61, 95% CI 0.37 to 0.85, t = 4.93, p < 0.001) and family and friends (0.73, 95% CI 0.50 to 0.96, t = 6.13, p < 0.001). There was no effect of disclosure of PrEP use to parents on PrEP adherence mediated through PrEP reminders from household members or from family and friends (Table 3). The posthoc assessment showed that AGYW who disclosed PrEP use to their parents and lived with them and those who did not live with them did not differ in their PrEP adherence from those who did not disclose PrEP use to their parents, except at month six, those who disclosed to their parents and did not live with them had an increase of TFV-DP concentration of 159 fmol/punch (95% CI 6 to 312, t = 2.04, p = 0.04) compared to those who did not disclose to them (Table 4).

**Table 3:** The effect of disclosure of PrEP use on PrEP adherence mediated through PrEP support among AGYW who perceived generally well supported by adults

Mediators	Increase in DBS TFV-DP concentration					
	Household members help remember			Family and Friends help remember		
	fmol/punch (95% CI)	Z test	P value	fmol/punch (95% CI)	Z test	P value
Month three adherence^a^						
Indirect effect	14 (− 24, 53)	0.73	0.47	10 (− 32, 52)	0.49	0.63
Direct effect of disclosure	116 (− 18, 250)	1.70	0.09	120 (− 15, 255)	1.74	0.82
Total effect of disclosure to parents^b^	130 (2, 250)	1.99	0.05	130 (2, 250)	1.99	0.48
Month six adherence^a^						
Indirect effect	34 (− 18, 87)	1.29	0.20	21 (− 34, 77)	0.75	0.45
Direct effect of disclosure to parents	143 (− 26, 312)	1.66	0.10	156 (− 15, 327)	1.79	0.07
Total effect of disclosure to parents^b^	177 (15, 340)	2.14	0.03	177 (15, 340)	2.14	0.03

*AGYW*: Adolescent girls and young women, *DBS TFV-DP* tenofovir-diphosphate concentrations in dried blood spots, *CI* confidence interval

^a^Adjusted for site and age

^b^The total effect (the regression coefficient without the mediator in the model) of disclosure of PrEP use on adherence is the sum of direct effect and indirect effect

**Table 4:** Disclosure of PrEP use by month three and housing with parents at month three on PrEP adherence at months three and six

	Increase in DBS TFV-DP concentration							
		Month three adherence^a^				Month six adherence^a^		
	Average (95% CI)	fmol/punch (95% CI)	T test	P value	Average (95% CI)	fmol/punch (95% CI)	T test	P value
Disclosed to parents and live with parents (n = 84)	431 (372, 490)	90 (− 20, 199)	1.60	0.11	308 (238, 379)	50 (− 92, 193)	0.69	0.49
Disclosed to parents but does not live with parents (n = 143)	472 (392, 554)	74 (− 42, 191)	1.26	0.21	461 (344, 578)	159 (6, 312)	2.04	0.04
Did not disclose to parents (n = 108)	397 (308, 485)	Ref			333 (223, 442)	Ref		

*DBS TFV-DP* tenofovir-diphosphate concentrations in dried blood spots, *CI* confidence interval

^a^Adjusted for site and age

## Discussion

Most AGYW in this open-label PrEP study chose to take oral PrEP and disclosed PrEP use to at least one person, most often parents, sexual partners, and friends. Of note, an AGYW’s decision to disclose PrEP use is not straightforward as she has to weigh the risks and benefits before disclosing [25, 45]. The high disclosure rates suggest the AGYW in this study decided the benefits of PrEP disclosure outweighed the risks, and those who did not disclose may have felt that there was no need to disclose or it posed a risk [25, 45]. One of the strengths of our study was it quantitively evaluated the relationship between PrEP disclosure and adherence at two times points (month three and again at month six). We hypothesized that AGYW would have higher adherence at both months three and six with disclosure but did not find this to be the case; we found no impact of disclosure of PrEP use on adherence at either timepoints. This lack of impact was the same regardless of the relationship type. With regard to support, more than half of the AGYW in our sample reported they felt generally well-supported by adults in their life. In the context of strong general support from adults, we found disclosure of PrEP use to parents was associated with increased adherence compared to AGYW who did not disclose PrEP use to their parents. This association was not dependent on living with parents. The amount of increased adherence was equal to taking about one additional pill per week, on average [46]. Parents have a strong influence from childhood to adolescence and our study provides evidence that PrEP disclosure to parents among AGYW who perceive that adults are generally supportive improves AGYW’s health-promoting PrEP adherence behavior.

Our study findings were similar to a previous PrEP demonstration study in South Africa that found by 6 months after initiating PrEP, 58% of AGYW (ages 16 to 25 years) disclosed PrEP use to their parents, 58% disclosed to partners, and a higher percentage (81%) disclosed to their friends [40]. However, only young women ≤ 18 years of age who disclosed to their parents were 6.8 times more likely to have high adherence at 6 months compared to those who did not disclose to a parent, but the study did not assess the reasons why. Our findings suggest that this difference may be because AGYW ≤ 18 years of age who disclosed to a parent felt well-supported by the adults in their life. In addition, in a study that explored the moderating effect of family dynamics in South Africa, peer adherence support for ART adherence had a positive impact in well-functioning families and a negative impact in dysfunctional families [28].

We found disclosure of PrEP use, especially to parents’,was associated with increased reminders to take PrEP by the household members. This finding is supported by previous research of focus group discussions among AGYW in a PrEP implementation study in Kenya and South Africa, where PrEP disclosure was associated with PrEP support in the form of reminders to take PrEP during the first 3 months after starting PrEP [25]. In particular, the mother’s support was important for integrating PrEP into their daily routine. Similarly, in a PrEP demonstration project in Tanzania and South Africa, AGYW reported disclosure of PrEP use resulted, for the most part, in gaining partner support in the form of reminders to take PrEP daily, but for some, it was a negative experience with having to respond to their partner’s resistance and anger [47]. Further, AGYW tended to initially disclose to supportive maternal figures (mothers, aunts, and older sisters) to gain PrEP support (i.e., explain frequent clinic visits and the PrEP pills) and as a form of respect, consistent with regional social norms. We hypothesized that the effect of PrEP disclosure on adherence was partially mediated through reminders from those to whom they disclosed PrEP use but did not find this to be the case. Despite the positive association between disclosure to parents and reminders, there was no change in the relationship between disclosure and adherence, meaning PrEP reminders were not on the pathway between disclosure and adherence. More research is needed to better understand the relationship between disclosure of PrEP use and adherence to PrEP. Our study had a few limitations. First, questions on reminders to take PrEP were not asked separately for parents, sexual partners, or friends. The lack of specificity by relationship type may have contributed to the lack of evidence of an association between reminders to take PrEP and PrEP adherence despite substantial evidence of disclosure to parents in the context of supportive adults resulting in increased adherence. Similarly, social support questions referred to the general term ‘adults’ and not by relationship types. Consequently, it was not possible to accurately assess whether parental PrEP support (i.e., through PrEP reminders) was in the causal pathway between PrEP disclosure to the parents and adherence. Given the likely differential influence of parents, sexual partners, and friends, it will be important in future research to collect general social support, disclosure, and PrEP support data for each type of relationship (parents, sexual partners, and friends) to tease out the independent contributions by each relationship type. Further, qualitative research strongly suggests that maternal support plays a significant role in PrEP and ART adherence for adolescents [25, 26, 47]. Hence, questions should differentiate between disclosures to maternal and paternal figures and their social and PrEP support.

## Conclusions

This open-label PrEP study in South Africa and Zimbabwe underscored that when African AGYW have supportive adults in their lives, disclosure to parents leads to increased PrEP adherence with AGYW taking about one pill more a week, on average compared to those who did not disclose. PrEP interventions that help identify supportive relationships and effectively communicate PrEP use to the parents or guardians, partners, and friends may help normalize PrEP use and improve adherence by African AGYW, and therefore PrEP effectiveness in this important population. Future research is needed to understand the pathways between a supportive environment, disclosure to specific relationships, and PrEP support by specific types of relationships to improve PrEP adherence.

## Data Availability

The data that support the findings of this study are available from the corresponding author upon reasonable request.
